# Pregnane Steroids from the Leaves of *Melia Azedarach* and Apoptotic Activity against T47D Cells

**DOI:** 10.31557/APJCP.2021.22.6.1967

**Published:** 2021-06

**Authors:** Martha Ervina, Hadi Poerwono, Retno Widyowati, Hideaki Otsuka, Katsuyoshi Matsunami, Sukardiman Sukardiman

**Affiliations:** 1 *Department of Pharmaceutical Sciences, Faculty of Pharmacy, Universitas Airlangga, Surabaya, Indonesia. *; 2 *Department of Pharmaceutical Biology, Faculty of Pharmacy, Widya Mandala Catholic University, Surabaya, Indonesia. *; 3 *Faculty of Pharmacy, Yasuda Women’s University, Yasuhigashi, Asaminami-ku, Hiroshima, Japan. *; 4 *Department of Pharmacognosy, Graduate School of Biomedical and Pharmaceutical Sciences, Hiroshima University, Japan. *

**Keywords:** Apoptosis, breast cancer, Melia azedarach, T47D, cytotoxicity

## Abstract

**Objective:**

Nature has provided us with many pharmaceutical resources so far. Breast cancer shows an increasing trend in the world for the last decade and becomes one of five leading causes of death. Among the plants, *Melia azedarach* L. has been used widely in traditional medicine for many ailments including breast cancer. Following our previous findings that the ethyl acetate fraction was the most active cytotoxic fraction against T47D cells, we aimed to isolate the cytotoxic compounds and further elucidate their apoptotic mechanisms.

**Methods:**

The compounds were isolated through a series of chromatography with cytotoxicity evaluations. Identification of the isolated compounds was achieved by intensive spectroscopic analysis such as NMR, MS, and IR spectra. Cytotoxicity was evaluated by MTT method using doxorubicin as a reference compound. The expression of apoptosis-related factors was quantified by flow cytometry and immunocytochemistry.

**Results:**

Two isomers of pregnane steroids with molecular weight 330.2087 (C_21_H_30_O_3_) were isolated from the EtOAc extract. Spectroscopic analysis revealed the structures as 17-ethylene-3,4-dihydroxy-14-methyl-18-norandrostene-16-one (1) and 17-ethylene-3,4-dihydroxy-5-pregnene-16-one (2), respectively. These compounds showed moderate cytotoxicity (IC_50_ 172.9 and 62.2 µg/mL, respectively) comparable to doxorubicin (IC_50_ 3.08 µg/mL). The execution of apoptosis may be related to the increase of the ratio of BAX/bcl-2 of the cells.

**Conclusion:**

The EtOAc fraction of *Melia azedarach* L. leaves and the isolated 5-pregnene-16-one steroids are promising reagents for breast cancer treatment by introducing apoptosis to tumor cells. However, further researches are required to highlight its safety and usage in vivo.

## Introduction

The increase of breast cancer incidence and its high mortality over the past decade has raised urgent concerns as a global burden of the life-threatening disease (WHO, 2021). Besides the effort on their early prognosis, the effective and selective chemotherapies, which prevents the resistance and severe side effect, are still urgent to discover. Natural products composed a large amount of recent-day of active pharmaceutical ingredients, most notably for cancer chemotherapy. Moreover, Tariq et al. (2017) reviewed 199 plant species (78 families including Meliaceae family) being used traditionally for cancer treatment and reported the relatively high rate of terpenoids class in these plant materials.

Tamoxifen (competitive estrogen) is used in combination with other treatments in several stages of breast cancer, which points out the role of the endocrine hormone in breast cancer therapy (Traboulsi et al., 2016). Moreover, apoptosis (programmed cell death) as an anticancer mechanism has a specific role in preventing the promotion, progression, and relapsing of cancer. *M. azedarach* has been demonstrated its activity to various tumor cell lines and the presence of various steroids (Wu et al., 2009; Ervina et al., 2020). Their safety against normal cell has been observed using the vero cell line (Srinivasan et al., 2015). Previous research obtained Melia’s extract and compounds which derived from any parts of it, have cytotoxic, intrinsic, extrinsic and cell arrest apoptosis pathways against some cells line (Kim et al, 1994; Tada et al., 1999; Akhihisa et al., 2013; Kikuchi et al., 2013; Zhang et al., 2005; Kim et al., 2012; He et al., 2010; Tang et al., 2012; Wu et al., 2009; Jafari et al., 2013). Though only limited experiments use breast cell line or search deeply on their mechanism, The Melia’s seed extract in combination with cancer drug has been reducing adenocarcinoma mammary tumors of CH3-mice (Sumarawati et al., 2017). In addition, the preliminary research showed the presence of steroid group compounds from the leaves and ethyl acetate fractions and exhibited cytotoxic activity against T47D cells (Ervina et al., 2020). Thus, this work purpose to continue the isolation process of the bioactive steroids compound from ethyl acetate fraction with cytotoxic guidance and obtain the apoptosis mechanism of the isolates. The research is important to discover the *M. azedarach* activity as nature source for effective and selective phytochemical for chemotherapy. 

## Materials and Methods

*Materials*

The leaves of *M. azedarach* L. were collected in October 2017 from Materia Medica, Batu, Indonesia (7.8673° S, 112.5199° E, ± 875 asl with average temperature ± 20 – 25°C). The specimen was documented at the Pharmacognosy Laboratory, Department of Pharmaceutical Sciences, Faculty of Pharmacy, Universitas Airlangga as Ma011017 (Ervina et al., 2020).

*General Experimental Procedures*

The open column chromatographies (CC) were used Silica gel 60 (E. Merck, Darmstadt, Germany) and ODS (reversed-phase) CC (Cosmosil 75C18-OPN, Nacalai Tesque, Kyoto, Japan; F = 35 mm, L = 350 mm), respectively. HPLC was performed on an ODS column (Inersil ODS, GL Science, Tokyo, Japan; F = 10 mm, L = 250 mm, 2.5 mL/min), pumped with JASCO PU-1580 intelligent and JASCO RI-930 intelligent detector. 1H and 13C-NMR spectra (spectrometer Jeol JNM-ECS400 at 400 MHz) were recorded, with TMS as an internal standard. IR spectra were measured with Perkin Elmer Spectrum One FT-IR spectrophotometer. The molecular weight and formula were obtained with positive ion HR-ESI-MS LTQ Orbitrap XL mass spectrometer (Thermo Fisher Scientific, Waltham, MA, USA). 

*Extraction and Isolation of the compounds*

The leaves were determined, deposited, prepared as described by Ervina et al., (2020). The ethyl acetate fraction (ME 81.2 g) was subjected to the Diaion HP-20 CC and eluated with MeOH, MeOH-CHCl_3_ (20:1) and acetone. The subfraction MeOH and MeOH-CHCl_3_ (ME1, 63.9 g) was evaporated and continue subjected on silica gel with CHCl_3_-MeOH (100:0, 20:1, 15:1, 10:1, 7:1, 5:1, 3:1, 2:1 and 0:100) to yield ME11-ME19. Meanwhile obtained of their cytotoxic bioactive subfraction, the ME11-ME19 separated further with ODS CC (Cosmosil 75 C18-OPN), with MeOH-H_2_O (1:9, 2:8, 3:7, 4:6, 5:5, 6:4, 7:3, 8:2, 9:1, 10:0) and acetone. This produced subfraction ME111-ME1111 to ME191-ME1911. Compounds 1 (23.5 mg) and 2 (40.1 mg) were isolated from ME127; while compound 3 (6.2 mg) was separated from ME114 with acetone 60% and 30%, respectively. Compound 1 and 2 proceed to apoptosis and structure elucidation, while compound 3 was not since IC_50_’s >500 µg/mL and limited quantity.

*Cytotoxicity test against T47D breast cancer cells*

The T47D cell was prepared followed as Ervina et al., (2020) with blank control of media, DMSO and doxorubicin as the standard reference. The percentage of viable cell and IC_50_ analysis was made with excel (linear regression of log concentration).

*Apoptosis Analysis by Flow Cytometry, and Antibody Staining of p53, BAX, bcl-2 immunocytochemistry*

The apoptosis induction of the isolates was obtained by flow cytometry and immunocytochemistry expression of p53, BAX and bcl-2. The cells are cultured in RPMI medium with FBS 10% and fungasol 0.1%. The method as described by Mutiah et al., (2017) with Doxorubicin as the reference standard. The Propidium Iodide reagent and PI-Annexin V were used for apoptosis analysis with a flow cytometer. The immunocytochemistry with the primary and secondary antibodies technique was used. They consisted of streptavidin, DAB, and hematoxylin reagents. The cells that express these proteins will be colored brown and counted with a light microscope. The results determined as percentage cells expressing the p53, BAX, and bcl-2.

## Results

*The cytotoxic of the subfractions and isolates*

Table 1 presents the cytotoxic results of the subfractions and isolates. The ME11 and ME12 subfractions exhibited low IC_50_ of 95.29±3.26 and 119±5.74 µg/mL, sequentially. This result guided to further isolation among the subfractions. The ME11 and ME12 were the subfraction resulted with CHCl_3_ and CHCl_3_-MeOH (1:0; 20:1) which more non polar to others. Furthermore, compounds 1 and 2 were isolated from ME127; which displayed four peaks of ME127A-ME127D. Among those peaks, it was compound 1 and 2 whose quantity enough to procced for cytotoxic test. The compound 3 resulted IC_50_ >500 µg/mL, while others gave promising IC_50 _172.91±3.57 and 62.15±1.37 µg/mL. The subfraction of ME11 resulted higher IC_50_ than its compound; might suppose of ME11’s other isolates activity. Since of IC_50 _compound 3 >500 µg/mL; thus, not proceed further.

*Identification of Compounds*

**Figure 1:**
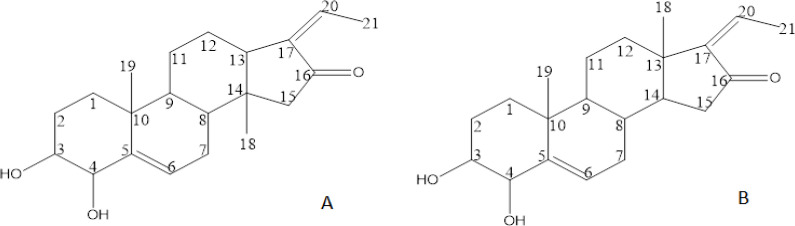
Structure of Compounds 1 (A) 17-ethylene-3,4-dihydroxy-14-methyl-18-norandrostene-14-methyl-16-one and 2 (B) 17-ethylene-3,4-dihydroxy-5-pregnene-16-one

The isolates were observed as purple spot with H_2_SO_4 _spray reagent. DBE (Double Bond Equivalents) resulted both have 7 double bonds (the steroid rings equivalents to four double bonds), so the isolates have three more unsaturated bonds. The 1H and 13C of the isolates are listed in Table 2, while their structure was described in Figure 1.

The compounds are resulted purple spot with H_2_SO_4 _spray reagent. They are isomers position form with molecular formula of C_21_H_30_O_3_ as determined by HR-ESI-MS at m/z of 330.2087 [M+Na]+ (calculated for C_21_H_30_O_3_Na: 353.2087). Their 1H NMR signals represent of three methyl, two oxygenated methine, three methyl, six methylene and seven methine protons signals. Lactone moiety and ethylene substituent were also indicated at C16 and C17. Compound 1 was obtained as a white amorphous powder. The IR spectrum implied the presence of carbonyl (1645.60 cm-1), hydroxy groups (3404.54 cm-1), and C=C (1718.60 cm-1), C-O bond (1205.71 and 1070.67 cm^-1^). The 1H-NMR spectrum (Table 2) represents signals of three methyl protons at δ 1.24 (3H, s), 1.06 and 1,87; two oxygenated methine protons at 3.42 (m, 1H), and 4.06 (s, 1H). The quaternary carbon were observed at C5, C10, C14 and C16’s Lactone moiety (δC 144.78, 38.87, 44.41 and 208.56 ppm, respectively). Compound 2 was obtained as a yellowish amorphous powder. The IR spectrum compound 2 implied the presence of carbonyl (1642.14 cm^-1^), hydroxy groups (3349.74 cm^-1^), and C=C (1712.40 cm^-1^). The 13C-NMR spectrum of compound 2 was most similar to compound’s 1 (Table 2) with slightly difference δ ± 1-2 ppm, while the 1H-NMR and COSY spectrum obtained different signals pattern (Table 2). The compound 2 represents signals of three methyl protons at δ 1.22 (s, 3H), 0.92 (s, 3H) and 2,02 (d, J=7,1 Hz, 3H); two oxygenated methine protons as hydroxyl substituent at 3.42 (m, 1H), and 4.04 (s, 1H). The quaternary carbon was observed at C5, C10, C13 and C16’s Lactone moiety (δC 145.00, 38.57, 40.52 and 210.94 ppm, sequentially). The HMBC of compound 2 was observed relation among C18 with C17; while C18 compound’s 1 with C6 and C17. 

*The apoptosis results of the isolates*

**Figure 2.**
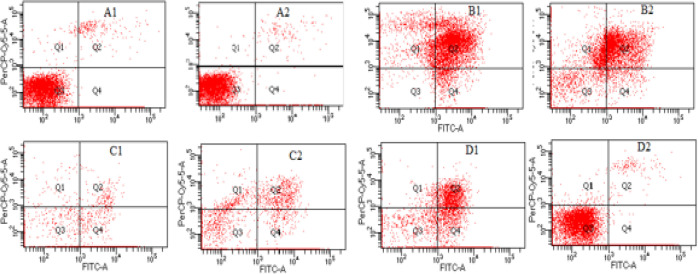
Flowcytometer with PI-annexin Staining Results of the ME127A, 127C and Doxorubicin for A = control cells, B = doxorubicin, compounds C = 1, D= 2, 1= 24 and 2 = 48 hours, phases cell Q1= necrosis, Q2 = late apoptosis, Q3 = viable cells, Q4 = early apoptosis

**Figure 3:**
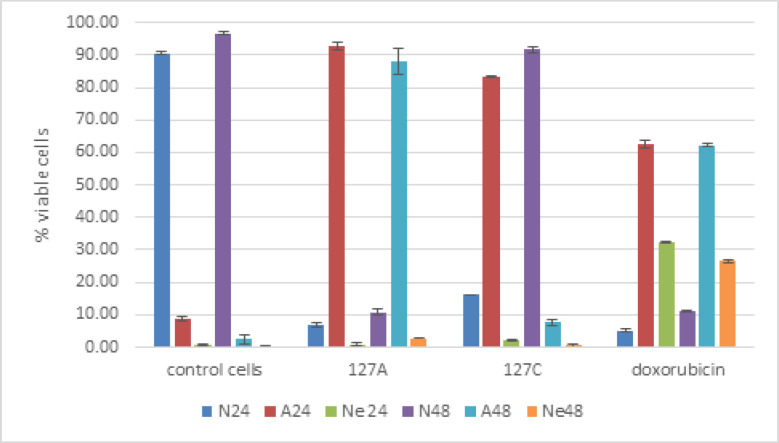
Flowcytometer Histogram Results of the Control (N), Apoptosis (A) and Necrosis (N) Cells for 24 and 48 hours of the Compounds 1, 2 and Doxorubicin

**Figure 4:**
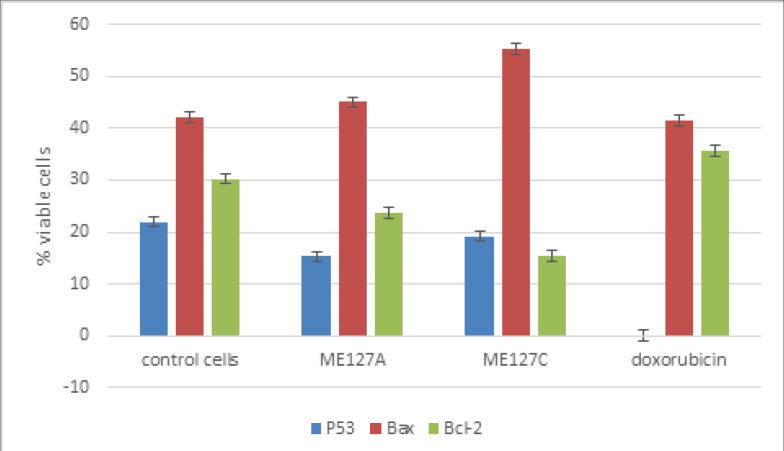
The Histogram of Microscopic Count of the p53, BAX and bcl-2 Immunocytochemistry with Antibody Reagents and Hematoxylins Staining Results of the Compounds 1, 2 and Doxorubicin

The compounds showed apoptosis induction as presents in Figures 2, and 3. The control cells were observed T47D without treatment, which was almost 100% of the viable cells. Meanwhile the treatments of compounds displayed reduction of the viable cell to 5-6% for compound 1, and doxorubicin, while 16% for compound 2. The treatments have higher percentage of apoptosis cell and lower of necrosis cells than the doxorubicin. The reference doxorubicin exhibited higher both percentage of apoptosis and necrosis cells. Moreover, Figure 2 is also described distribution of the cell treatment of the compounds and doxorubicin, which were mostly on Q2 (late) and Q4 (early). This represented the cell undergo dominantly late than early apoptotic stage. The microscopic observation obtained apoptotic bodies, elongated cell shapes, and shrink cytoplasm, distancing among the cells, and reducing the number of the treatments and doxorubicin viable cells. Furthermore, the immunocytochemistry of the treatment resulted increasing on BAX, reducing on bcl-2 and no significant difference on p53 (Figure 4).

**Table 1:** The MTT Cytotoxic Results of the Subfraction and Isolates

Sub fractions (ME)	IC_50_ (µg/mL)*
11	95.29±3.26^b^
12	119.17±5.74^b^
13	209.07±5.26^c^
14	>500^f^
15	294.62±3.02^d^
16	343.40±11.57^de^
17	379.69±5.84^e^
18	342.95±4.60d^e^
19	395.27±19.79^e^
Compound 1	172.91±3.75^bc^
Compound 2	62.15±1.37^b^
Compound 3	>500^f^
Doxorubicin	3.08±0.20^a^

* different superscript letter means Anova statistically significant (p < 0.05) between the means in the same column

**Table 2:** ^13^C and ^1^H NMR Data of Compounds 1 and 2 (CDOD3, 400 MHz, J in Hz).

No	signal (δ, ppm)			
	^13^C		^1^H	
	1	2	1	2
1	37.49	36.88	2,19 (s, H)	1.88 (s, H)
2	25,80	26.00	2.06 (t, *J* = 2.5, 1H); 1.96 (m)	1,97 (m)
3	73.81	73.95	3.42 (m, 1H)	3.42 (dt, *J* = 11.9, 4.0 Hz, 2H)
4	78.52	78.64	4.06 (s, 1H)	4.04 (d, *J* = 3.4 Hz, 2H)
5	144.78	145.00	-	-
6	127.99	128.19	5.63 (dd, 1H)	5.61 (dd, *J* = 4.8, 2.2, 2H)
7	32.9	32.35	2.13 (dd, *J* = 11.3, 6.2, 1H)	
8	37,39	32.92	1.85 (d, 3H)	2.13 (m, *J* = 7.3, 2H)
9	51.88	51.92	1.45 (m)	0.96 (s,1H); 3.28 (t, *J* = 2.9, 1.5, 2H)
10	38,87	38.57	-	-
11	21.54	21.68	2.39 (d, *J* = 8.4, 1H)	1.06 (d, *J* =4.2, 1 H)
12	32.02	37.68	1.56 (m); 1.13 (t, 2H)	1.85 (t); 1.57 (m),
13	38.41	40.52	1.69 (d)	-
14	44.41	51.55	-	2.10 (d, *J* = 5.1, 1H)
15	51.52	44.39	2.02 (s, 1H), 2.19 (d, *J* = 7.1, 1H)	2.14 (d, *J* = 2.83, 2H)
16	208.56	210.94	-	-
17	149.45	149.77	-	-
18	16.1	20.01	1.06 (s, 3H)	0.92 (s, 3H)
19	21.54	21.48	1.24 (s, 3H)	1.22 (s, H)
20	130.9	131.99	6.47 (q, *J* = 7.5 Hz, 1H)	5.78 (q, *J* = 7.3 Hz, H)
21	13.51	14.33	1.87 ((d, *J* = 7.3, 3H)	2.02 (d, *J* = 7.3, 3H)

## Discussion

*M. azedarach* (Meliaceae) is widely distributed in almost one third of the world, and has been used traditionally to overcome various health problems both locally and systemically (Sultana et al., 2014; Sharma & Paul, 2013). It has been used as cytotoxic in vitro to in vivo against many cells line culture. As its anti-breast cancers, it showed against SK-BR-3, and MCF-7 (Jafari, et al., 2013). The previous research also resulted, its ethyl acetate fraction activity against the T47D cell line and a positive correlation between total sterol to its cytotoxic activity (Ervina et al., 2020). This work had a purpose to continue the isolation with bioassay guided, identification of active compounds and their apoptosis mechanism. 

The *M. azedarach* leaves ethanol extract was subjected to series of column chromatography over Diaion, silica gel, ODS, and semipreparative HPLC to yield two steroids (Figure 1). The use of many systems in the separation process was to eliminate the chlorophyll, lipid acid and many inert organic compounds from the fractions and subfractions, meanwhile improving the purity of the isolates. Prior to the isolation process, the cytotoxicity bioassay guided on their subfraction is listed in Table 1. The IC50 cytotoxicity resulted from the ME11 and ME12 subfractions were lower than others. This means the potential bioactive compounds were supposed on ME11 or ME12 than in the other subfractions. Furthermore, the ME114 and ME127 were proceed to the purification process with semipreparative HPLC. 

The HPLC optimized separation yielded the compounds 1 and 2 from ME127. Their IC50 were lower than another (Table 1), this means the compounds 1 and 2 were more active against T47D than compound 3. Thus, the mechanism obtained on compounds 1 and 2. Meanwhile, comparing IC50 among the extract to isolates also represent the purification process would improve their cytotoxic activity (compare to doxorubicin; from ethanolic extract, ethyl acetate fraction to subfractions and isolates, respectively).

The ME114A were analyze as flavonoid glycoside but not further proceed for apoptosis since obtained lower cytotoxic activity, meanwhile the compounds 1 and 2 were proceeds to apoptosis test mechanism. All isolates TLC-H2SO4 spot, the IR profile, MS fragmentation and molecular formula, NMR and the physical properties data were compared to the previous finding database. The compound 3 has yellow spot, while the compounds 1 and 2 gave purple spot. The compounds were identified as Quercetin 3*-O-β-D*-galactopyranosyl-(1-6)-*β-D*-galactopyranoside and two isomers of modified pregnane steroid. The flavonoid glycoside was identified by submitting the NMR signal analysis to the database (JEOL NMR) and comparing to the previous data (Hirayama et al., 2013). Meanwhile, the compound 1 and 2 obtained many similarities on NMR signals as reported previously (Wu et al., 2009). 

The compounds have steroid 3*β*,4*β*-Dihydro-5-pregnene-16-one skeleton with ethylene on C-17 and methyl substituent on C-16. Both were isomers which have a similar molecular formula C_21_H_30_O_3_. It was determined from the HR-EIMS positive mode with ion peak at m/z 353.2087 [M+Na]. Both have similar fragmentation pattern with slightly difference on their abundance, the m/z compound 1 showed higher abundance at 313.22 while compound 2 was at 353.21. Their IR also have observed hydroxyl at 3,404.54; 3,349.74, double bonds at 1,718.60; 1712.40, and carboxyl at 1,642.14; 1,645,60, respectively. The compounds structures were elucidated base on 1H, 13C, COSY, HMQC and HMBC data analysis. The overlapping 1H signal at 1.3 – 2.25 ppm made difficult to obtain HMQC relation between carbon and proton respectively. Both 13C presented of two hydroxyl, three methyls (sp3), six methylenes (sp2) and seven methines (sp), one carbonyl and ethylenes. The main differences between compounds 1 and 2 are that the signal on C12, C13, C14, and C15 which highlighted the isomer position of methyl substituent on C13 to C14, respectively (Table 2). The COSY NMR obtained hydroxyl on ortho-position on C3, and C4, while the HMBC showed the carbonyl, the ethylene and methyl on D-ring. The DBE (double bond equivalent) resulted from three double bonds more to the steroid ring. These data analyses represented of compound 2 as 5-pregnene-16-one steroid with hydroxyl on C3, and C4; ethylene on C17; while modified of C13-methyl substituent of compound 2 to C-14 on compound 1 (Figure 1). The HMBC data of the compounds highlighted the correlation among atom in the structures. That the ethylene at C17 has corelation to proton at C15, and C21 at both compounds. The isomer position of methyl between the compounds were also observed from HMBC’s of C18. C18’s compound 1 has correlation to proton C14, and C15; while compound 2 has corelation to C17 (Figure 1). From the databases, the compounds shared similar molecular formulas to progesterone steroid (C_21_H_30_O_3_) but the NMR spectra did not support the finding. The 13C signals were many identical with 5-pregnene-16-one (Wu et al. 2009). Furthermore, they also reported the cytotoxicity of the steroids from *M. azedarach* leaves against A549, H460 and U251, though not presented the cytotoxic data of compound 5-pregnene-16-one. This finding gives improved scientific data to the related compounds. 

Moreover, the apoptotic induction results of the isolates and doxorubicin are as shown in Figures 2, and 3. Apoptosis was performed at the IC_50_ concentration on 24 and 48 hours. The prolonged time test schemed to the observed the significant difference among them. Though, a significant difference has been observed on 24 hours already. The T47D control cell was observed the highest percentage of cells at 90.60% and 96.67%; and few cells undergo apoptosis and necrosis (Figure 2). In the treated cells showed significant apoptosis compared to control cells, even though both had different characteristics. The percentage apoptosis cells of compound 1 were more than compound 2 at 24 hours, while the necrotic cells of compound 2 was slightly higher; even though lower than Doxorubicin. Furthermore, at 48 hours, the viable (normal) cells of compound 2 increased. It’s number close to the percentage of control cells, while the compound 1’s resulted 4% increasing. This seems, cell have regulated to the compounds, especially to compound 2. The decrease in the percentage of apoptotic and necrotic cells was observed on both treatments, but the contrary noticed on the number of necrotic cells of compound 1. Meanwhile in the doxorubicin, the apoptotic and necrotic cells were almost the same both at 24 and 48 hours. This means doxorubicin, the anthracycline chemotherapy substances, showed no selective apoptosis to necrosis mechanism. The unwanted inflammation reaction would observe as necrosis processes occur. Thus, this research highlighted the advantage of compounds 1 and 2 compare to doxorubicin at 24 hours. 

The intrinsic mechanism via improving BAX/bcl-2 ratio was supposed interfere in this apoptosis mechanism. The microscopic observed of apoptotic bodies in the treatment cells, and counted for the increasing of the BAX/bcl-2 ratio as Sumarawati et al. (2013) previous result, though no significant different on p53. The qualitative observation cell distribution among the quadrans of flow cytometer results (Figure 2) presented the early and late apoptotic mechanism of the compounds and doxorubicin. The increasing of BAX/bcl-2 treatments cells were supported the qualitative early apoptotic stage (Q4) description; while for the late and mid apoptotic process had not been observed in this work. 

The compounds were difference on Methyl substituents. The compound 1 has it on C14 while compound 2 has on C13. It seems Methyl substituents on C13 influence its cytotoxic or apoptosis activities of the isolates. The estrogen steroid has been already known its role in the development and proliferation of breast cancer. Thus, the tamoxifen (antagonist estrogen) binds to estrogen receptor-α and genetically inhibits the proliferation, while induces apoptosis in breast cancer (Li et al., 2017). Vundru et al., (2013) observed the phenomenon of cell death by *β*-sitosterol. They related the induction of apoptosis with depolarization of the mitochondrial membrane potential and an increase in the protein BAX/bcl-2. The compound 2 main structure has more similarity to β-sitosterol, while compound 1 was analyzed as its isomer. Both isolate structures were considered as derivate of progesterone, with hydroxyl on C4, ketone and ethylene groups. From the previous study it was obtained the role of C13-methyl substituent to 3ERT (human estrogen receptor-α), while Motamed et al., (2020) found the influence of progesterone on MCF-7 apoptosis. It increased the BAX p53; caspase 3 and 9; while reducing the bcl-2. In addition, Creamer et al., (2020) literally found the pregnane X receptor (PXR, NR1I2) role in the breast tissue. The activation of PXR has the main function in apoptosis, and develops in acquired resistance to chemotherapeutic substances. Though many works on PXR role in tumorigenesis are still required.

## Author Contribution Statement

Study conception and design ME, HP and S.; methodology ME, S, KM; formal analysis ME, RW, HO, KM; investigation ME; resources ME, S, KM, HO; writing—original draft preparation ME; writing—review and editing ME and all; visualization ME; supervision S, HP RW, HO, KM; project administration ME; funding acquisition S, KM. All authors have read and agreed to the published version of the manuscript.

## References

[B1] Akihisa T, Pan X, Nakamura Y (2013). Limonoids from the fruits of Melia azedarach and their cytotoxic activities. Phytochem.

[B2] Creamer BA, Sloan SNB, Dennis JF (2020). Review associations between pregnane X receptor and breast cancer growth and progression. Cells.

[B3] Ervina M, Poerwono H, Widyowati R, Matsunami K, Sukardiman 2020) Bio-selective hormonal breast cancer cytotoxic and antioxidant potencies of Melia azedarach L. wild type leaves. Biotech Rep.

[B4] He Y, Wang J, Liu X (2010). Toosendanin inhibits hepatocellular carcinoma cells by inducing mitochondria-dependent apoptosis. Planta Med.

[B5] Hirayama C, Ono H, Meng Y, Shimada T, Daimon T (2013). Flavonoids from the cocoon of Rondotia menciana. Phytochem.

[B6] Jafari S, Saedinia S, Hajimehdipoor H (2013). Cytotoxic evaluation of Melia azedarach in comparison with Azadirachta indica and its phytochemical investigation. DARU J Pharma Sci.

[B7] Kikuchi T, Pan X, Ishii K (2013). Cytotoxic and apoptosis-inducing activities of 12-o-acetylazedarachin B from the fruits of Melia azedarach in human cancer cell lines. Biol Pharm Bull The Pharm Soc Japan.

[B8] Kim HM, Oh GT, Han SB (1994). Comparative studies of adriamycin and 28-deacetyl sendanin on in vitro growth inhibition of human cancer cell lines. Arch Pharmacol Res.

[B9] Kim HW, Kang SC (2012). The toxicity and anticancer activity of the hexane layer of Melia azedarach L var japonica Makino’s bark extract. Toxicol Res.

[B10] Li W, Shi X, Xu Y (2017). Tamoxifen promotes apoptosis and inhibits invasion in estrogen positive breast cancer MCF 7 cells. Mol Med R.

[B11] Motamed HR, Shariati M, Ahmadi R, Khatamsaz S, Mokhtari M. (2020). The apoptotic effects of progesterone on breast cancer (MCF-7) and human osteosarcoma (MG-36) cells. Physiol Int.

[B12] Mutiah R, Widyawaruyanti A, Sukardiman. (2017). Calotroposid A: a glycosides terpenoids from calotropis gigantea induces apoptosis of colon cancer. Asian Pac J Cancer Prev.

[B13] Srinivasan V, Panneerselvam R, Gunasekaran S, Palani S (2015). Neuro-protective activity of ethanolic extract of Melia azadirachta against H2O2 induced toxicity in vero cell line. Int J App Biol Pharm Tech.

[B14] Sharma D, Paul Y (2013). Preliminary and pharmacological profile of Melia azedarach An overview. J Appl Pharm Sci.

[B15] Sultana S, Asif HM, Akhtar N, Waqas M, Rehman SU (2014). Comprehensive review on ethnobotanical uses, phytochemistry and pharmacological properties of Melia azedarach Linn. Asian J Pharm Res Health Care.

[B16] Sumarawati T, Israhnanto, Fatmawati D (2017). Anticancer mechanism of Melia azedarach, doxorubicin and cyclosphamide combination against breast cancer in Mice. Bangladesh J Med Sci.

[B18] Traboulsi T, Ezzy ME, Gleason JL, Mader S (2016). Antiestrogens: structure activity relationships and use in breast cancer. J Mol Endocrinol.

[B19] Tada K, Takido M, Kitanaka S (1999). Limonoids from fruit of Melia toosendan and their cytotoxic activity. Phytochem.

[B20] Tang XL, Yang XY, Kim YC (2012). Protective effects of the ethanolic extract of Melia toosendan fruit against colon cancer. Indian J Biochem Biophys.

[B21] Vundru SS, Kale SK, Singh RP. (2013). β- sitosterol induces G1 arrest and causes depolarization of mitochondrial membrane potential in breast carcinoma MDA-MB-231 cells. BMC Compl Alt Med.

[B22] World Health Organization (2021). Cancer: Fact sheets.

[B23] Wu SB, Ji YP, Zhu JJ (2009). Steroid from the leaves Melia azedarach and their cytotoxic effects on human cancer cell lines. Steroids.

[B24] Zhang B, Feng Wang Z, Zhi Tang M, Liang Shi Y 2005) Growth inhibition and apoptosis-induced effect on human cancer cells of toosendanin, a triterpenoid derivative from Chinese traditional medicine. Invest New Drugs.

